# Open and remotely accessible Neuroplatform for research in wetware computing

**DOI:** 10.3389/frai.2024.1376042

**Published:** 2024-05-02

**Authors:** Fred D. Jordan, Martin Kutter, Jean-Marc Comby, Flora Brozzi, Ewelina Kurtys

**Affiliations:** FinalSpark, Rue du Clos 12, Vevey, Switzerland

**Keywords:** wetware computing, organoid intelligence, biocomputing, synthetic biology, AI, biological neural network, hybrot

## Abstract

Wetware computing and organoid intelligence is an emerging research field at the intersection of electrophysiology and artificial intelligence. The core concept involves using living neurons to perform computations, similar to how Artificial Neural Networks (ANNs) are used today. However, unlike ANNs, where updating digital tensors (weights) can instantly modify network responses, entirely new methods must be developed for neural networks using biological neurons. Discovering these methods is challenging and requires a system capable of conducting numerous experiments, ideally accessible to researchers worldwide. For this reason, we developed a hardware and software system that allows for electrophysiological experiments on an unmatched scale. The Neuroplatform enables researchers to run experiments on neural organoids with a lifetime of even more than 100 days. To do so, we streamlined the experimental process to quickly produce new organoids, monitor action potentials 24/7, and provide electrical stimulations. We also designed a microfluidic system that allows for fully automated medium flow and change, thus reducing the disruptions by physical interventions in the incubator and ensuring stable environmental conditions. Over the past three years, the Neuroplatform was utilized with over 1,000 brain organoids, enabling the collection of more than 18 terabytes of data. A dedicated Application Programming Interface (API) has been developed to conduct remote research directly via our Python library or using interactive compute such as Jupyter Notebooks. In addition to electrophysiological operations, our API also controls pumps, digital cameras and UV lights for molecule uncaging. This allows for the execution of complex 24/7 experiments, including closed-loop strategies and processing using the latest deep learning or reinforcement learning libraries. Furthermore, the infrastructure supports entirely remote use. Currently in 2024, the system is freely available for research purposes, and numerous research groups have begun using it for their experiments. This article outlines the system’s architecture and provides specific examples of experiments and results.

## 1 Introduction

The recent rise in wetware computing and consequently, artificial biological neural networks (BNNs), comes at a time when Artificial Neural Networks (ANNs) are more sophisticated than ever.

The latest generation of Large Language Models (LLMs), such as Meta’s Llama 2 or OpenAI’s GPT-4, fundamentally rely on ANNs.

The recent acceleration of ANN use in everyday life, such as in tools like ChatGPT or Perplexity combined with the explosion in complexity in the underlying ANN’s architectures, has had a significant impact on energy consumption. For instance, training a single LLM like GPT-3, a precursor to GPT-4, approximately required 10 GWh, which is about 6,000 times the energy a European citizen uses per year. According to a recent publication the energy consumption projected may increase faster than linearly (De Vries, 2023). At the same time, the human brain operates with approximately 86 billion neurons while consuming only 20 W of power (Clark and Sokoloff, 1999). Given these conditions, the prospect of replacing ANNs running on digital computers with real BNNs is enticing (Smirnova et al., 2023). In addition to the substantial energy demands associated with training LLMs, the inference costs present a similarly pressing concern. Recent disclosures reveal that platforms like OpenAI generate over 100 billion words daily through services such as ChatGPT as reported by Sam Altman, the CEO of OpenAI. When we break down these figures, assuming an average of 1.5 tokens per word—a conservative estimate based on OpenAI’s own tokenizer data—the energy footprint becomes staggering. Preliminary calculations, using the LLaMA 65B model (precursor to Llama 2) as a reference point, suggest energy expenditures ranging from 450 to 600 billion Joules per day for word generation alone (Samsi et al., 2023). While necessary for providing AI-driven insights and interactions to millions of users worldwide, this magnitude of energy use underscores the urgency for more energy-efficient computing paradigms.

Connecting probes to BNNs is not a new idea. In fact, the field of multi-unit electrophysiology has an established state of the art spanning easily over the past 40 years. As a result, there are already well-documented hardware and methods for performing functional electrical interfacing and micro-fluidics needed for nutrient delivery (Gross et al., 1977; Pine, 1980; Wagenaar et al., 2005a; Newman et al., 2013). Some systems are also specifically designed for brain organoids (Yang et al., 2024). However, their research is mostly focused on exploring brain biology for biomedical applications (e.g., mechanisms and potential treatments of neurodegenerative diseases). The possibility of using these methods for making new computing hardware has not been extensively explored.

For this reason, there is comparatively less literature on methods that can be used to reliably program those BNNs in order to perform specific input–output functions (as this is essential for wetware computing, not for biomedical applications). To understand what we need for programming of BNNs, it is helpful to look at analogous problem for ANNs.

For ANNs, the programming task involves finding the network parameters, globally denoted as S below, that minimize the difference L computed between expected output E and actual output O , for given inputs I , given the transfer function T of the ANN. This can be written as:

$L=f\left(O,E\right)$ , with $O=T\left(I,S\right)$

where f is typically a function that equals 0 when O=E .

The same equation applies to BNNs. However, the key differences compared to ANNs include the fact that the network parameters S cannot be individually adjusted in the case of BNNs, and the transfer function T is both unknown and non-stationary. Therefore, alternative heuristics must be developed, for instance based on spatiotemporal stimulation patterns (Bakkum et al., 2008; Kagan et al., 2022; Cai et al., 2023a,b). Such developments necessitate numerous electrophysiological experiments, including, for instance, complex closed-loop algorithms where stimulation is a function of the network’s prior responses. These experiments can sometimes span days or months.

To facilitate long-term experiments involving a global network of research groups, we designed an open innovation platform. This platform enables researchers to remotely perform experiments on a server interfaced with our hardware. For example, our Neuroplatform enhances the chances of discovering the abovementioned stimulation heuristics. It should be noted that, outside of the field of neuroplasticity, similar open platforms were already proposed in 2023 (O’Leary et al., 2022; Armer et al., 2023; Elliott et al., 2023; Zhang et al., 2023). However, to our knowledge, there are no platforms specifically dedicated to research related to biocomputing.

## 2 Biological setup

**Figure 1:**
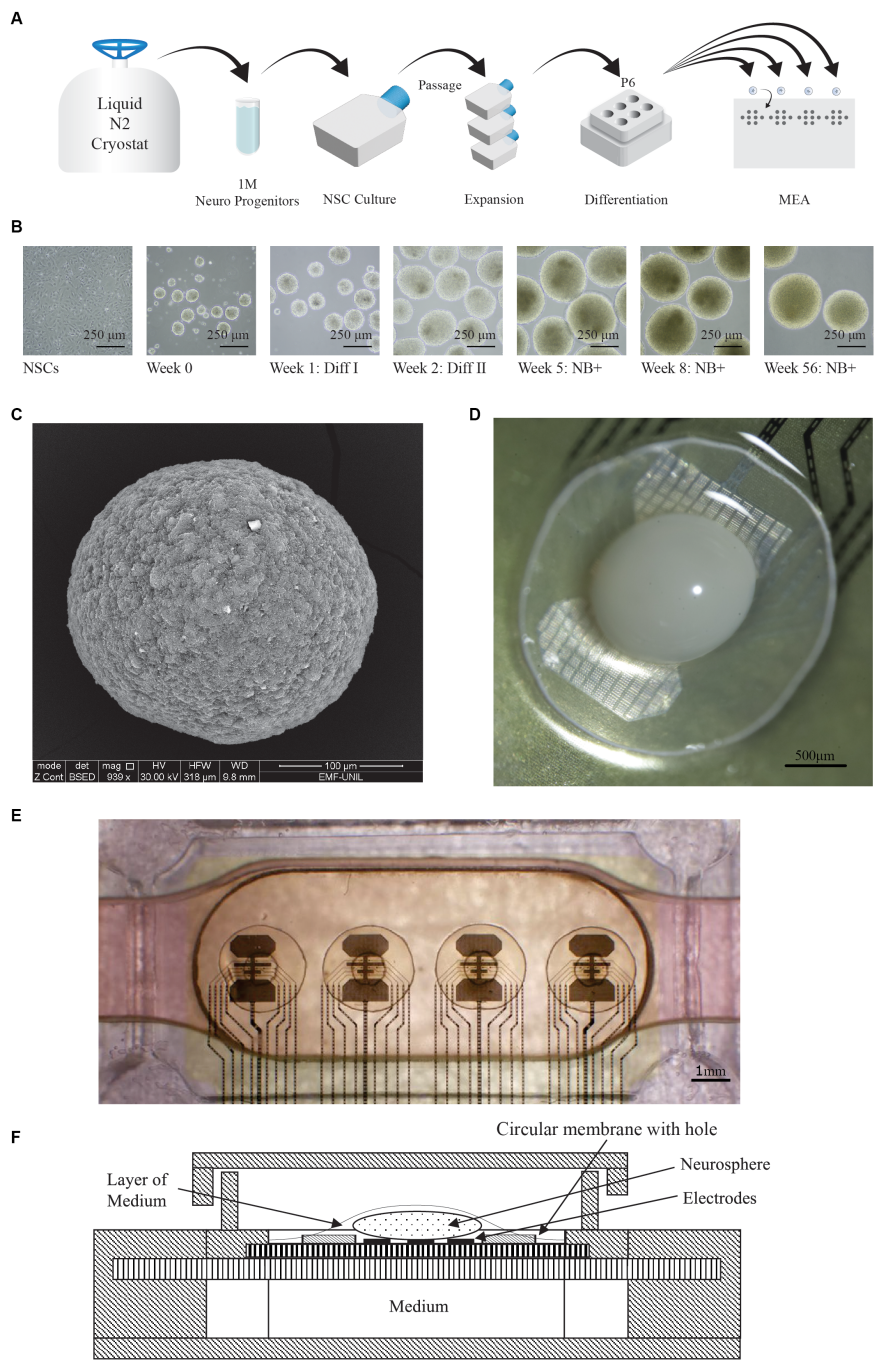
FO generation and MEA setup. **(A)** Protocol used for the generation of forebrain organoids (FO). Neural progenitors are first thawed, plated and expanded in T25 flasks. They are then differentiated in P6 dishes on orbital shakers, and finally manually placed on the MEA. **(B)** Representative images showing various stages of FO formation and differentiation, taken at different time points. The scale bar represents 250 μm. **(C)** Image of a whole FO taken with scanning electron microscope. The scale bar represents 100 μm. **(D)** Microscope view of the FO (in white) sitting on the electrodes of the MEA, and the membrane. The hole in the membrane is not visible on the picture since it is hidden by the FO. The scale bar represents 500 μm **(E)** Overview of the MEA, where the 32 electrodes are visible as 4 sets of 8 electrodes each. An FO is placed atop of each set of 8 electrodes, visible as a darker area. For each FO, the 2 circles correspond to a 2.5 mm circular membrane with a central hole. The scale bar represents 1 mm. **(F)** Cross-sectional view of the MEA setup, illustrating the air-liquid interface. The medium covering the FO is supplied from the medium chamber through the porous membranes.

The biological material used in our platform is made of brain spheroids [also called minibrains (Govindan et al., 2021), brain organoids (Qian et al., 2019), or neurospheres (Brewer and Torricelli, 2007)] developed from Human iPSC-derived Neural Stem Cells (NSCs), following the protocol of Prof. Roux Lab (Govindan et al., 2021). Based on the recent guidelines to clarify the nomenclature for defining 3D cellular models of the nervous system (Paşca et al., 2022), we can call those brain spheroids “forebrain organoids” (FOs). Generation of brain organoids from NSCs has been already described for both mouse (Ciarpella et al., 2023), and human models (Lee et al., 2020). Our protocol is based on the following steps: expansion phase of the NSCs, induction of the 3D structure, differentiation steps (using GDNF and BDNF), and maturation phase (Figures 1A,B). The Figure 1C is an image of the FO obtained using electronic microscope, it shows that it is a compact spheroid. The average shape of FOs obtained with this protocol are spheroids of a diameter around 500 μm (Govindan et al., 2021). Our experiments show that the FOs obtained can be kept alive in an orbital shaker for years, as previously demonstrated (Govindan et al., 2021).

Gene expression analysis of mature FOs vs. NSCs showed a marked upregulation of genes characteristic to neurons, oligodendrocytes and astrocytes in FOs compare to NSCs. More precisely, FOs expressed genes typically enriched in the forebrain, such as striatum, sub pallium, and layer 6 of motor cortex (Govindan et al., 2021). Pathway enrichment analysis of FOs vs. NSCs demonstrated activation of biological processes like synaptic activity, neuron differentiation and neurotransmitter release (Govindan et al., 2021).

At the age of 12 weeks, FOs contain a high number of ramified neurons (Govindan et al., 2021), and they are mature enough to be transferred to the electrophysiological measurement system (Figure 1A). In this setup, they have a life expectancy of several months, even with 24/7 experiments that include hours of electrical stimulations. This setup has a quick turnaround with occasional downtime – about 1 h – during organoid replacements. Therefore, the platform maintains a high availability for experiments.

## 3 Hardware architecture

### 3.1 Introduction

The remotely accessible hardware includes all the systems which are required to preserve homeostasis, monitor environmental parameters and perform electrophysiological experiments. These systems can be controlled interactively using our custom Graphical User Interface (GUI) or via Python scripts. All data is stored in a time-series database (InfluxDB), which can be accessed either via a GUI or via Python scripts. The users typically connect to the system using the Remote Desktop Protocol (RDP).

The platform is composed of several sub-systems, which can be accessed remotely via API calls over the internet, typically through Python.

### 3.2 Multi-Electrode Array (MEA)

Our current platform features 4 MEAs. The MEAs were designed by Prof. Roux’s Lab form Haute Ecole du Paysage, d’Ingénierie et d’Architecture (HEPIA) and are described in Wertenbroek et al. (2021). Each MEA can accommodate 4 organoids, with 8 electrodes per organoid (Figure 1E).

The MEA setup utilizes an Air-Liquid-Interface (ALI) approach (Stoppini et al., 1991), in which the organoids are directly placed on electrodes located atop of a permeable membrane (Figure 1D), with the medium flowing beneath this membrane in a 170 μL chamber. As a result, a thin layer of medium, created by surface tension, separates the upper side of the organoids from the humidified incubator air. This arrangement is further protected by a lid partially covering the MEA (Figure 1F). This ALI method enables a higher throughput and higher stability compared to submerged approaches, since no dedicated coating is required, and it is less prone to have the organoids detaching from the electrodes.

### 3.3 Electrophysiological stimulation and recording system

**Figure 2:**
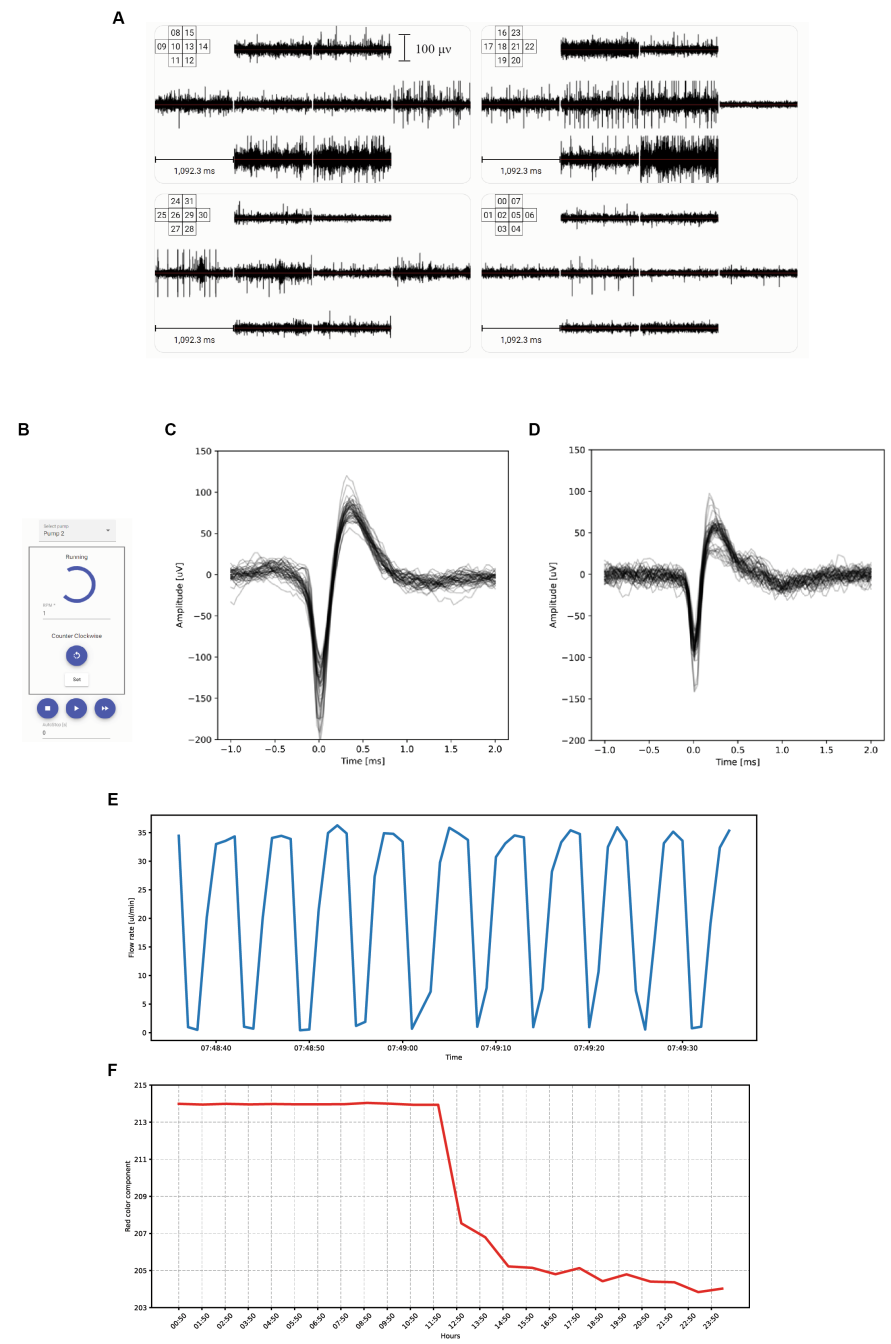
Recording system and user interface. **(A)** Electrical activity measured in μV over one second for each of the 32 electrodes. Each set of 8 electrodes records a different FO. **(B)** Graphical User Interface for manually controlling each of the microfluidic pumps. **(C)** Overlays of FO action potential recorded by the ALI system of the Neuroplatform. **(D)** Overlays of FO action potentials recorded with an MCS system. **(E)** Fluctuations of the flowrate of the medium within the microfluidic system, illustrating the cyclic variations induced by the peristaltic pump operating at 1 round per minute with 10 cams. **(F)** Temporal variations of the red component of the medium color, triggered by a sudden change in medium acidity, resulting in phenol red color change.

The electrodes in our system enable both stimulation and recording. The respective digital-to-analog and analog-to-digital conversions are performed by Intan RHS 32 headstages. Stimulations are executed using a current controller that ranges from 10 nA to 2.5 mA, and recordings are obtained by measuring the voltage on each electrode at a 30 kHz sampling frequency with a 16 bits resolution giving an accuracy of 0.15 μV. The headstages are connected to an Intan RHS controller, which in turn is connected to a computer via a USB port. The Figure 2A shows the electrical activity recorded for each of the 32 electrodes. It can be noticed that the recorded activity is different between each electrode. This difference comes from the facts that each set of 8 electrodes records a different FO and that for a given FO, electrodes record at a different location. This display is refreshed in real-time and also available 24/7 on our website at the URL https://finalspark.com/live/. We compared the recording characteristics of this ALI setup to MCS MEA (60MEA200/30iR-Ti) monitoring a submerged FO, using the exact same Intan system for voltage conversion. The overlays of an action potential recorded, respectively, with the ALI and submerged versions are shown in Figures 2C,D and show similar signal characteristics.

### 3.4 Micro-fluidics

**Figure 3:**
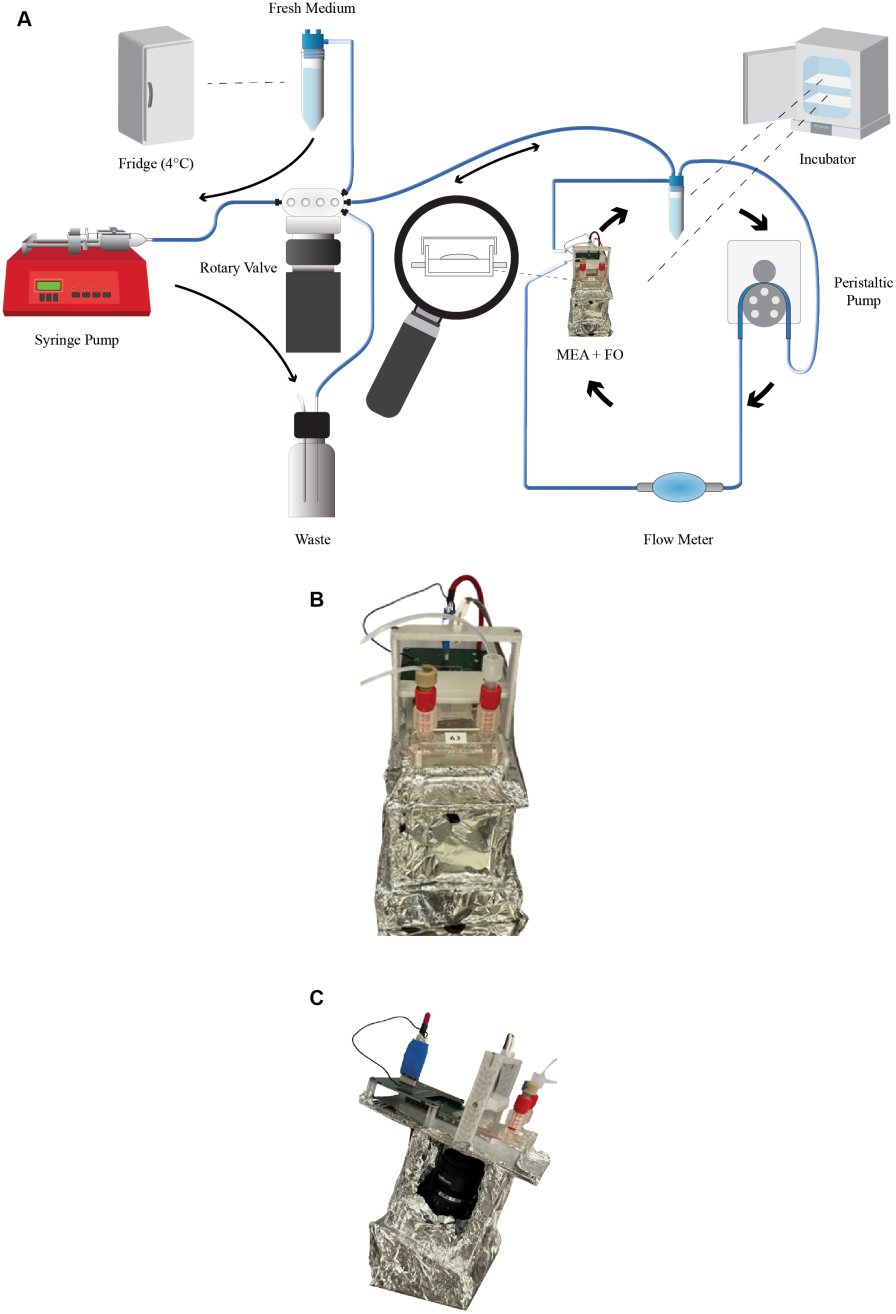
Microfluidics. **(A)** Microfluidic system illustrating the continuously operating primary system, which ensures constant flow in the medium chamber, and the secondary system responsible for medium replacing every 48 h. **(B)** Side view of the assembly, featuring the camera and the MEA. The entire assembly is enclosed with aluminum foil to ensure the lowest possible noise level. **(C)** Front view of the assembly, showing the intake and outtake of the microfluidic system, as well as the LED used during image capture.

To sustain the life of the organoids on the MEA, Neuronal Medium (NM) needs to be constantly supplied. Our Neuroplatform is equipped with a closed-loop microfluidic system that allows for a 24/7 medium supply. The medium is circulating at a rate of 15uL/min. The medium flow rate is controlled by a BT-100 2 J peristaltic pump and is continuously adjusted according to needs, for instance during experimental runs. The peristaltic pump is connected to the PC-control software using an RS485 interface, for programmed (i.e., in Python) or manual operations (Figure 2B). Additionally, Figure 3A depicts this microfluidic closed-loop circuit.

The microfluidic circuit is made of 0.8 mm (inside diameter, ID) tubing. Continuous monitoring of the microfluidic circuit and flow rate is achieved by using Fluigent flow-rate sensors, which connect to the Neuroplatform control center via USB. Data related to medium flow rate is stored in a database for later access. Figure 2E shows the cyclic variations in flow induced by the cams of the peristaltic pump.

A secondary microfluidic system is used to replace the medium in the closed-loop with fresh medium every 24 h, a process illustrated in Figure 3A. This replacement is fully automated through a Python script and performed in the following consecutive steps:

1. Set the rotary valve to select the path from the reservoir F50 to the syringe pump
2. Pump 2 mL of old medium using the syringe pump
3. Set the rotary valve to select the path from the syringe pump to the waste F50
4. Push 2 mL of old medium to the waste using the syringe pump
5. Set the rotary valve to select the path from the new medium in the F50 in the fridge to the syringe pump
6. Pump 2 mL of fresh medium using the syringe pump
7. Set the rotary valve to select the path from the syringe pump to the reservoir F50
8. Push 2 mL of fresh medium using the syringe pump

### 3.5 Cameras

Each MEA is equipped with a 12.3-megapixel camera that can be controlled interactively or programmatically (i.e., through a Raspberry Pi) for still image capture or video recording. The camera is positioned below the MEA, while illumination is provided by a remotely controlled LED situated above the MEA. Figures 3B,C illustrate this assembly (the aluminum wrapping is used in order to minimize the noise). This setup is particularly useful for detecting various changes, such as cell necrosis, possible organoid displacement caused by microfluidics, variations in medium acidity (using color analysis since our medium contains Phenol red), contamination, neuromelanin production (which can happen when uncaging dopamine), overflows (where the medium inadvertently fills the chamber above the membrane), or bubbles in the medium. For the latter two events, dedicated algorithms automatically detect these issues and trigger an alert to the on-site operator.

Changes of acidity, for example, can be detected by measuring the average color over a pre-defined window. Figure 2F shows the evolution of the medium’s red color component, with data points recorded hourly. The noticeable sudden drop is attributed to the pumping of medium with a slightly different acidity. This change in acidity results in a color alteration of the phenol red present in the medium.

### 3.6 UV light controlled uncaging

It is also possible to release molecules at specific timings using a process called uncaging. In this method, a specific wavelength of light is employed to break open a molecular “cage” that contains a neuroactive molecule, such as Glutamate, NMDA or Dopamine. A fiber optic of 1,500 μm core diameter and a numerical aperture of 0.5 is used to direct light in the medium within the MEA chamber. The current system, Prizmatix Silver-LED, operates at 365 nm with an optical power of 260 mW. The uncaging system is fully integrated into the Neuroplatform and can be programmatically controlled during experiment runs via our API (see section 5.3).

### 3.7 Environmental measurements

The environmental conditions are monitored within two incubators. In both incubators, the following parameters are recorded: CO2, O2 concentrations, humidity, atmospheric pressure and temperature. Door-opening events are also logged since they have a major impact on measurements. The primary purpose of this monitoring is to ensure that experiments are performed in stable and reproducible environmental conditions.

**Figure 4:**
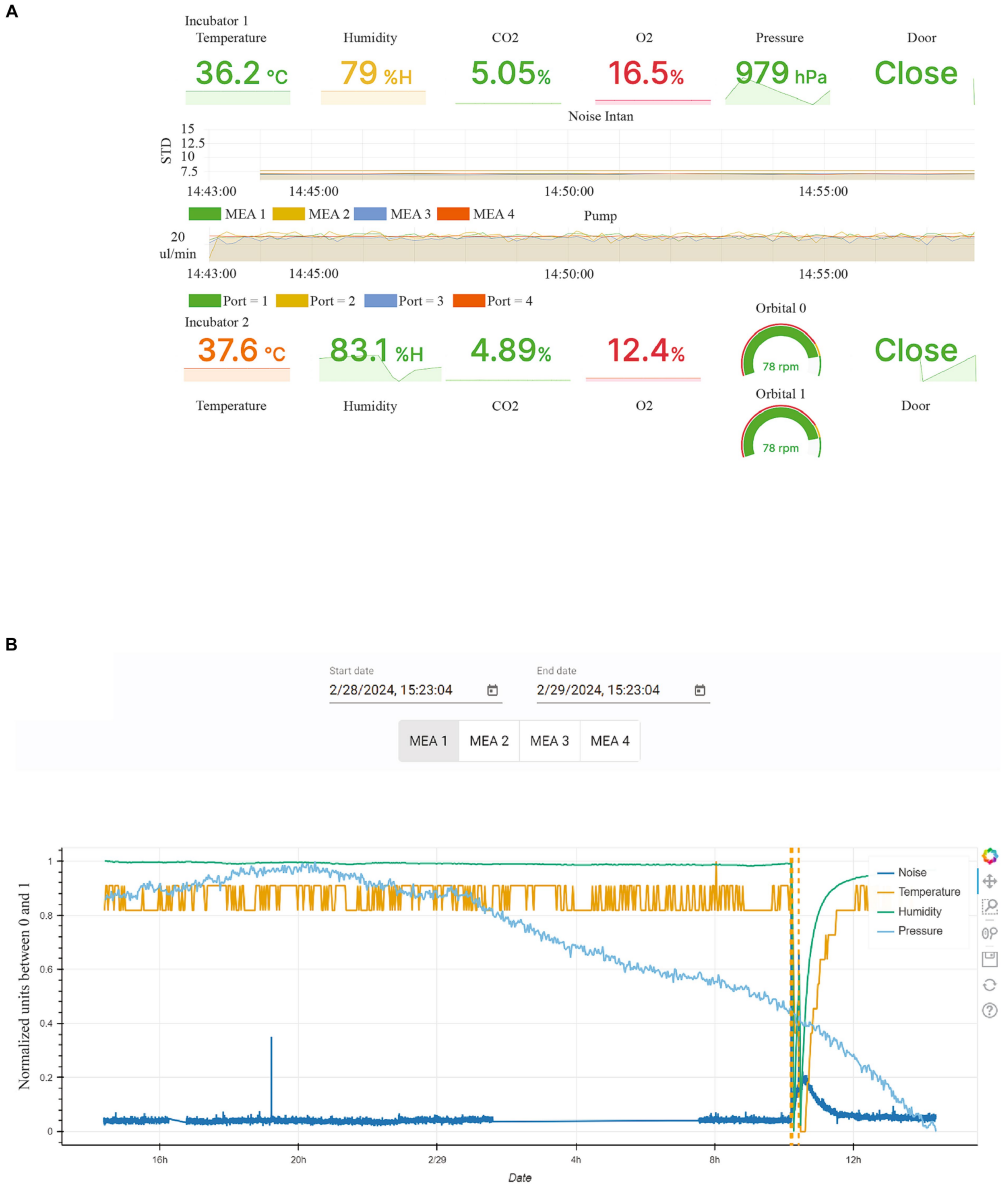
Graphic user interface to monitor critical parameters in the incubators. **(A)** Graphical User Interface displaying critical environmental conditions for the incubator 1, where electrophysiological experiments are performed, as well as the incubator 2, where FO are maintained on an orbital shaker. **(B)** The display shows environmental data for incubator 1 for specific time periods, extracted from the database, with door opening events displayed as dashed line. Noise, Temperature, humidity and pressure are indicated by different colored lines. The units of each measurement are normalized between 0 and 1 for the selected time interval.

All these parameters are displayed in real-time in a graphic interface showing both instant values as well as variations versus time of noise and flowrates (Figure 4A).

Incubator 1 houses the MEAs and the organoids used for electrophysiological experiments. In addition to the mentioned parameters, flowmeters are also utilized to report the actual flow rate of the microfluidic for each MEA, as depicted in the graph labelled “Pump” in Figure 4A. The system’s state is indirectly monitored through the noise level of each MEA, as shown in the graph labelled “Noise Intan” in Figure 4A. The noise level is calculated based on the standard deviation of the electrical signals recorded by the electrodes over a 30 ms period.

Incubator 2 houses the organoids which are kept in orbital shakers. Piezoelectric gyroscopes are used to measure the actual rotation speed of the orbital shakers.

Since all the data is logged in the database, it is also possible to access the historical measurements through a dedicated GUI (Figure 4B).

## 4 Software

### 4.1 General architecture

**Figure 5:**
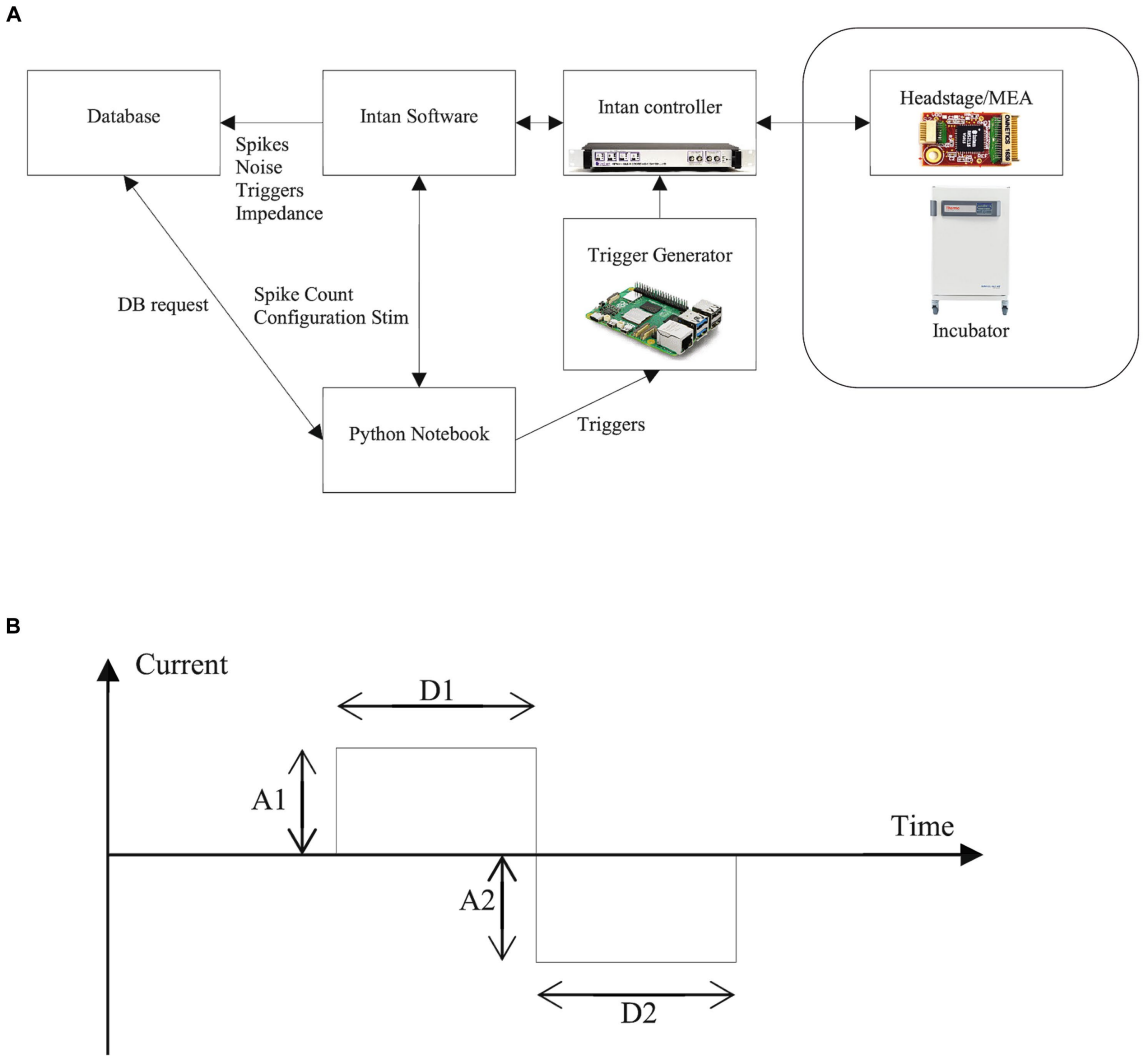
Software setup and electrical stimulation. **(A)** General architecture of the Neuroplatform. The Jupyter Notebook serves as the main controller, enabling initiation and reading of spikes, configuration stimulation signals and access to database via, e.g., Python **(B)** Parameters of the stimulation current: settings optimally these parameters can elicit spikes. Through the Python API, parameters that can be adjusted for the bi-phasic stimulation signals include the duration (D1) and amplitude (A1) of the positive current phase, and, respectively, D2 and A2 for the negative current phase. Additionally, the polarity of the biphasic signal can be reversed to start with a negative current.

The core of the system relies on a computational notebook which provides access to 3 resources (Figure 5A):

1. A database where all the information regarding the Neuroplatform system is stored
2. The Intan software running on a dedicated PC, which is used for:

- Recording the number of detected spikes in a 200 ms time window
- Setting stimulation parameters

3. A Raspberry Pi for triggering current stimulation according to stimulation parameters

### 4.2 Database

The Neuroplatform records monitored data 24/7 using InfluxDB, a database designed for time series. Other options are also available.

This database contains all the data coming from the hardware listed in Section 3.

The electrical activity of the neurons is also recorded 24/7 at a sampling rate of 30 kHz. To minimize the volume of stored data, we designed a dedicated process that focuses on significant events, such as threshold crossings that are likely to be due to action potentials (spikes). The following pseudo code illustrates the implemented approach:

- Each 1-min write buffer to database
- Each 33 μs
- For each electrode
- If, at time *t*, the voltage exceeds a threshold *T*
- Store (in buffer) 3 ms of data [*t*-1 ms, *t* + 2 ms]
- Each 3 s update T

Additionally, a timestamp corresponding to each detected event is also stored in the database, along with the maximum value of voltage during the 3 ms spike waveform recording.

The threshold *T* is computed directly from voltage values sampled each 33 μs, according to the following formula:


$$T=6*Mdn\left(\left\{\sigma_{i}\right\}\right)$$

Where $\sigma_{i}$ is the standard deviation computed over a set *i* of 30 ms consecutive voltage values, and $Mdn\left(\right)$ represents the median function computed over 101 consecutive $\left\{\sigma_{i}\right\}$ values. The use of the median reduces the sensitivity to outliers, which is typically caused by action potentials. In our current setup, a multiplier of 6 on the median has proven to be a good compromise for achieving reliable spike detection.

Besides electric tension data, the number spikes recorded per minute is also computed and stored in the database every minute by a batch process.

### 4.3 Recording electrical activity

As previously discussed, the communication among neurons is captured by the MEA and converted into a voltage signal sampled at 30 kHz. The Neuroplatform offers two basic access modes to the recorded neural activity:

1. Raw: raw sampling values.
2. Optimized: waveforms of the raw signal near neuronal spikes, available directly from the database.

In addition to the aforementioned features, the Neuroplatform offers even more advanced methods. For instance, it includes counting spikes over a fixed time period of 200 ms following stimulation, with a 10 ms delay suppressing the stimulation artifact.

From a technical perspective, accessing the number of spikes can be accomplished in two different ways:

- Retrieving the number of spikes per minute from the database
- Through direct communication with the PC managing the Intan controller for spike count

The second approach is required when the stimulation protocol demands real-time responsiveness. This is typically the case for certain closed-loop strategies. For instance, closed-loop stimulation strategies have been deployed in primary cortical cultures for effective burst control (Wagenaar et al., 2005a,b) and for goal-directed learning (Samsi et al., 2023).

### 4.4 Syntax for stimulations

Programmatically stimulating the FO on the Neuroplatform is accomplished by sending an electrical current to the MEA electrodes. The electrical current profile can be parameterized in a variety of ways, which is partly shown in Figure 5B. These parameters and controls include:

- Basic shape of stimulation signal:

o Bi-phasic
o Bi-phasic with interphase delay
o Tri-phasic

- Stimulation duration and intensity:

o Positive (A1) and negative (A2) electrical current intensity (typical 1uA, ranging from 0.1uA to 20uA)
o Duration of positive (D1) and negative (D2) stimulation currents

- Stimulation triggers

o Single start
o Table with collection of start triggers
o Pulse trains

- MEA electrodes

send_stim_param (electrodes, params)

## 5 Examples of electrophysiological experiments

To demonstrate the effectiveness of the Neuroplatform, the following sections will provide an overview of several experiments conducted on the Neuroplatform at FinalSpark’s Laboratories in Vevey, Switzerland.

### 5.1 Modification of spontaneous activity

The spontaneous electrical activity of the FO can be represented by the concept of “Center of Activity” (CA) (Bakkum et al., 2008) which is defined as a virtual position C on the MEA described by:


$$C=\frac{\sum_{k=1}^{8}F_{k}\cdot \left(X_{k},Y_{k}\right)}{\sum_{k=1}^{8}F_{k}}$$

Where $\left(X_{k},Y_{k}\right)$ define the spatial position of the 8 electrodes and $F_{k}$ is the number of spontaneous spikes detected. The interest of the concept of CA is that its position provides statistical information about the average location of the activity over the surface of the FO. The ability to change the position of the CA is interesting because it also shows the ability to memorize information in the state of the FO.

The coordinates of the CA can be modified using a high frequency stimulation. In the following experiment we use the following protocol:

1) Compute the CA using the number of detected spikes over 500 ms
2) Goto 1,100x
3) Perform a 20 Hz stimulation during 500 ms using a bi-phasic current (negative first) of 2 μA of 200 μS, for both phases, on one electrode
4) Wait 1 s
5) Goto 5,100x

**Figure 6:**
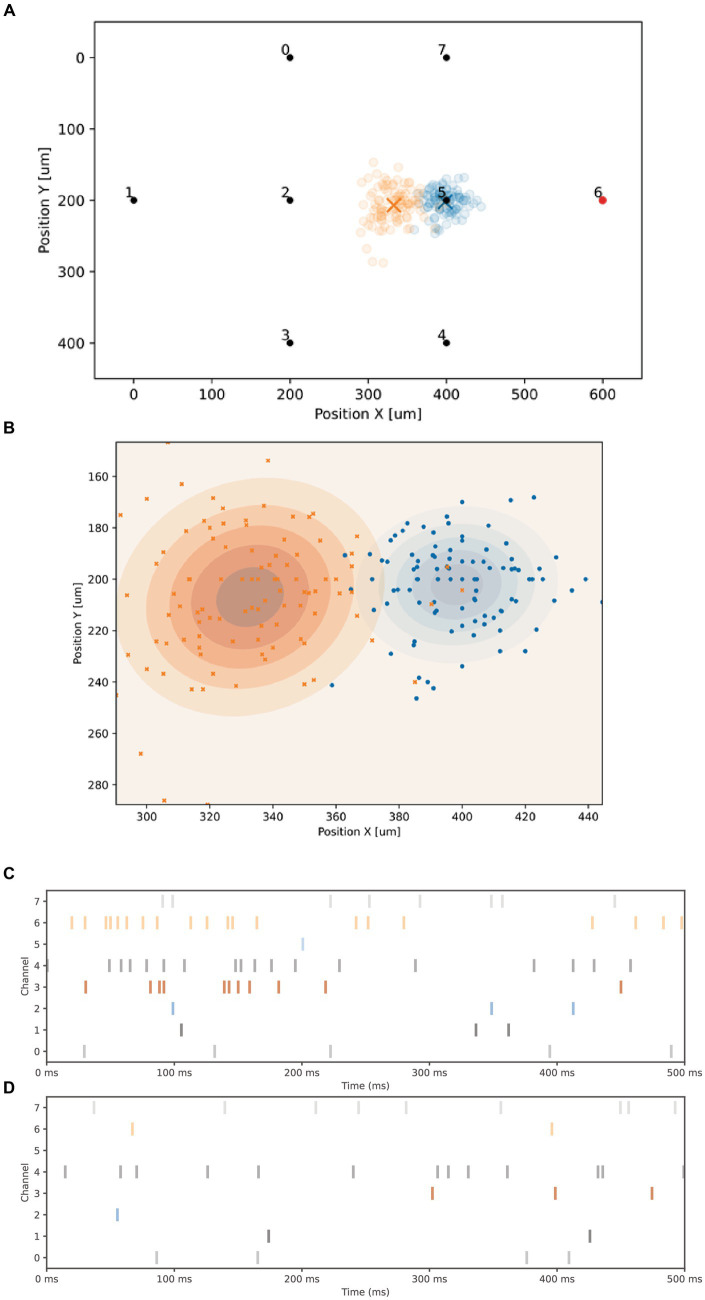
Center of activity modification. **(A)** Graph showing the 2D layout of the 8 electrodes, the X and Y axis are normalized units showing the spatial coordinates of the electrodes. All electrodes can be used for both stimulation and reading. A 20 Hz stimulation signal is applied to electrode 6. The 100 blue circles represent the positions of the Center of Activity (CA) before 20 Hz stimulation, while the 100 red circles indicate the positions after the stimulation. The cross mark the average position. **(B)** A closer look at the two groups of CA. **(C)** Timestamps depicting the spontaneous activity over 500 ms for each of the 8 electrodes before the high-frequency stimulation. **(D)** Spontaneous activity observed after the high-frequency stimulation, showing a lower activity of electrodes 6, 4 and 3, compared to **(C)**.

Figure 6A displays the 100 measured positions of the CA corresponding to the spontaneous activity before the 20 Hz stimulation in blue, and after the high-frequency stimulation in red (the average position is indicated by a cross). A close-up is shown in Figure 6B. The timestamps of the spontaneous activity, before and after stimulation, are presented in Figures 6C,D, respectively. Each graph shows one example of the 100 records of 500 ms used to compute the CA location (showing a decrease of spontaneous firing activity of electrodes 3, 4 and 6). A noticeable shift in the average position (shown by a cross) of the CA can be observed before and after the high-frequency stimulation (as seen in Figure 6A), indicating a change of state of the biological network. A classifier based on a simple logistic regression is employed to predict if the network has received the 20 Hz stimulation. In this particular experiment, the classification accuracy, computed from the confusion matrix, is 95.5%.

The Neuroplatform allows users to perform both the experimental part (including stimulation and reading operations) and the visualization of the CA displacement within the same Python source code. The 500 ms 20 Hz signal is generated directly by the Python source code shown below. The first trigger.send instruction sends the trigger for the stimulation on a specific electrode and time.sleep pauses the execution for 50 ms.
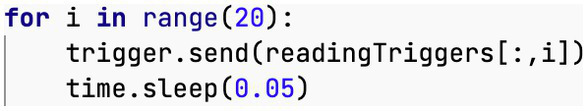


Despite the common perception of Python as being less than ideal for real-time signal processing due to its inherent latency, our empirical data reveals a time accuracy of under 1 ms (on an Intel Xeon CPU E5-2690 v2 @ 3.00GHz), a level of precision that is satisfactory for the generation of tetanic signals.

### 5.2 Optimization of stimulation parameters

In this example, the objective is to identify the set of stimulation parameters that can elicit the maximum number of action potentials within 200 ms after a stimulation.

**Figure 7:**
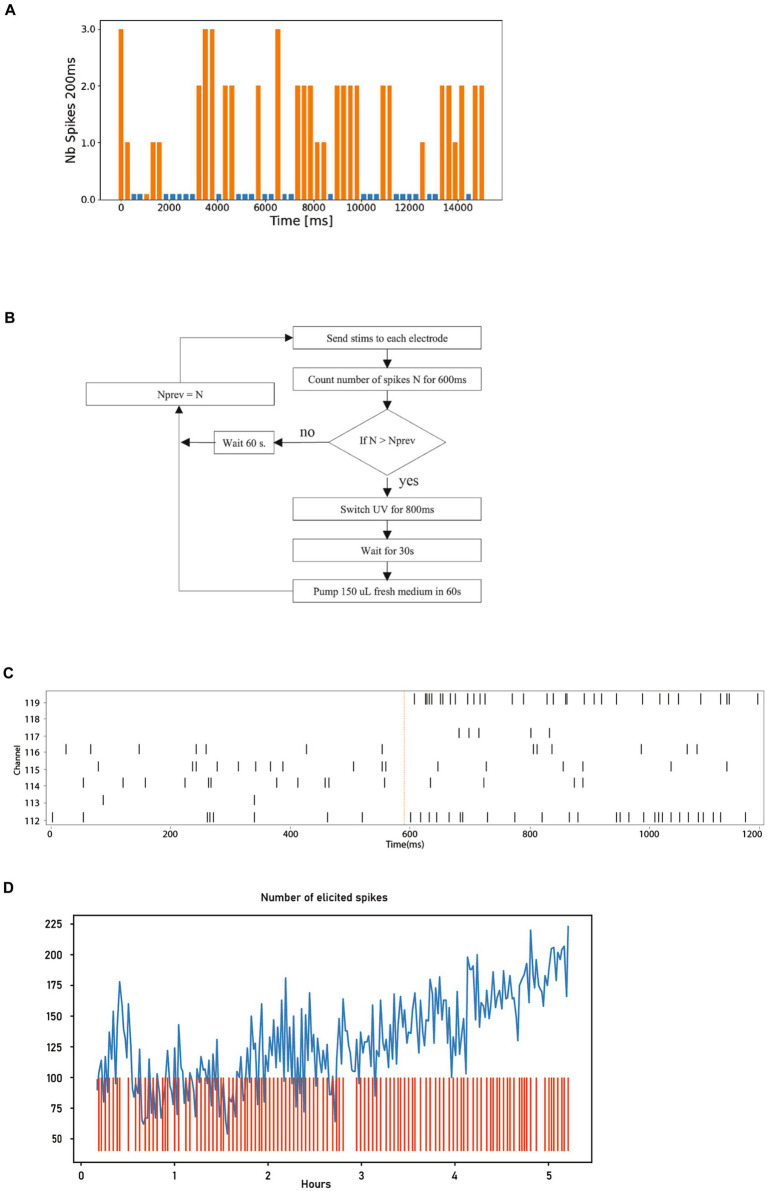
Neural activity stimulation and dopamine uncaging. **(A)** Graph depicting the number of spikes recorded over 250 ms. The spike counts in orange were measured following a stimulation, while those in blue were measured during periods without stimulation. For clarity in visualization, a small bar is displayed even when no spikes are detected. **(B)** Diagram illustrating the different steps involved in the closed-loop uncaging process of dopamine, which is repeated 240 times. **(C)** Timestamps of action potentials from the 8 electrodes before and after stimulation (shown as red line), showcasing the elicited spikes. **(D)** Graph displaying the number of elicited spikes over the 240 steps of the closed-loop (in blue) alongside the activation events of the UV light source (red).

Depending on the FOs, their composition, and maturity, only specific combinations of electrodes and parameters can elicit spikes. In our experiment, we use an 8-electrode MEA and cycle through several stimulation signal parameters as shown in Figure 7A. Consequently, we need to test a total of 342 different parameter-electrode combinations. The following pseudo code illustrates the Python script used in this experiment.

1) For each set of stimulation parameters
2) For each stimulation electrode
3) For each recording electrode
4) During 15 s, every 250 ms
5) Decide between stimulating, or recording spontaneous activity, with a 50% probability
6) Record number of spikes during 200 ms

The aim of probabilistic stimulation and no stimulation in step 5 is to evaluate the difference between elicited and spontaneous spikes in a way that ensures there is no bias.

The bar chart in Figure 7A displays a segment of the experimental results. It shows a 15-s recording from a single electrode, corresponding to one execution of step 4 in the pseudo code above. Each bar represents the spike count during a 200 ms period, repeated every 250 ms. The orange bars in this plot are the result of the parameters selected in step 1 of the pseudo code. The blue bars represent no-stimulation periods, thus corresponding to the spontaneous activity of the neurons.

From Figure 7A, we can see that this particular combination of electrode and parameters reliably elicits responses.

In practice, the Python script can also be used to automatically display the 342 graphs similar to Figure 7A, allowing the operator to select the optimal set of parameters. Additionally, it can compute a scalar metric to characterize the “efficiency” of the parameters, and automatically identify the optimal parameters.

An example of a parameter maximization metric is given in the equation below. Let us denote $\mu_{r}$ and $\mu_{s}$ the average number of spikes recorded spontaneously or after a stimulation, respectively, and $\sigma_{r}$ and $\sigma_{s}$ as their standard deviations. The following metric is used:


$$m=\frac{\left|\mu_{r}-\mu_{s}\right|}{\max\left(\sigma_{r},\sigma_{s}\right)}$$

The set of parameters that maximize this metric can then be utilized to perform other experiments requiring elicited spikes, such as investigating the effect of pharmacological agents on a biological network’s ability to react quickly to stimulation.

### 5.3 UV light-induced uncaging of molecules

‘Uncaging’ is a pivotal technique in cellular biology, enabling the precise control of molecular interactions within cells (Gienger et al., 2020). It involves the use of photolabile caged compounds that are activated by specific light wavelengths, releasing bioactive molecules in a targeted and timely manner. This method is particularly valuable for studying dynamic processes in neural networks and intracellular signaling, offering real-time insights into complex biological mechanisms.

Our Neuroplatform is equipped with all necessary components to perform uncaging. In this example, we investigate closed-loop stimulation, where dopamine is used to reward the network when more spikes are elicited by the same stimulation. The release of the dopamine is achieved through the uncaging of CNV-dopamine using the UV system described in section 3.6.

Figure 7B shows the flow chart of the closed-loop uncaging process. The optimal stimulation parameters are first found using the technique shown in 5.2 (in this case, a current of 4uA, biphasic with 100uS per phase), which is sent successively to each of the 8 electrodes with a delay of 10 ms between each electrode.

Figure 7C shows the response timestamps of the 8 electrodes for a period of 1,200 ms, 600 ms before and after the stimulation. The stimulation event is indicated by the vertical red line. It is interesting to observe that in this particular case, most of the elicited spikes originate from 2 electrodes, specifically electrode 112 and electrode 119.

The Python source code implementing the closed-loop process illustrated in Figure 7B is provided below. We would like to highlight here how concise the code is. With only 13 lines of code, the entire closed-loop process has been implemented.
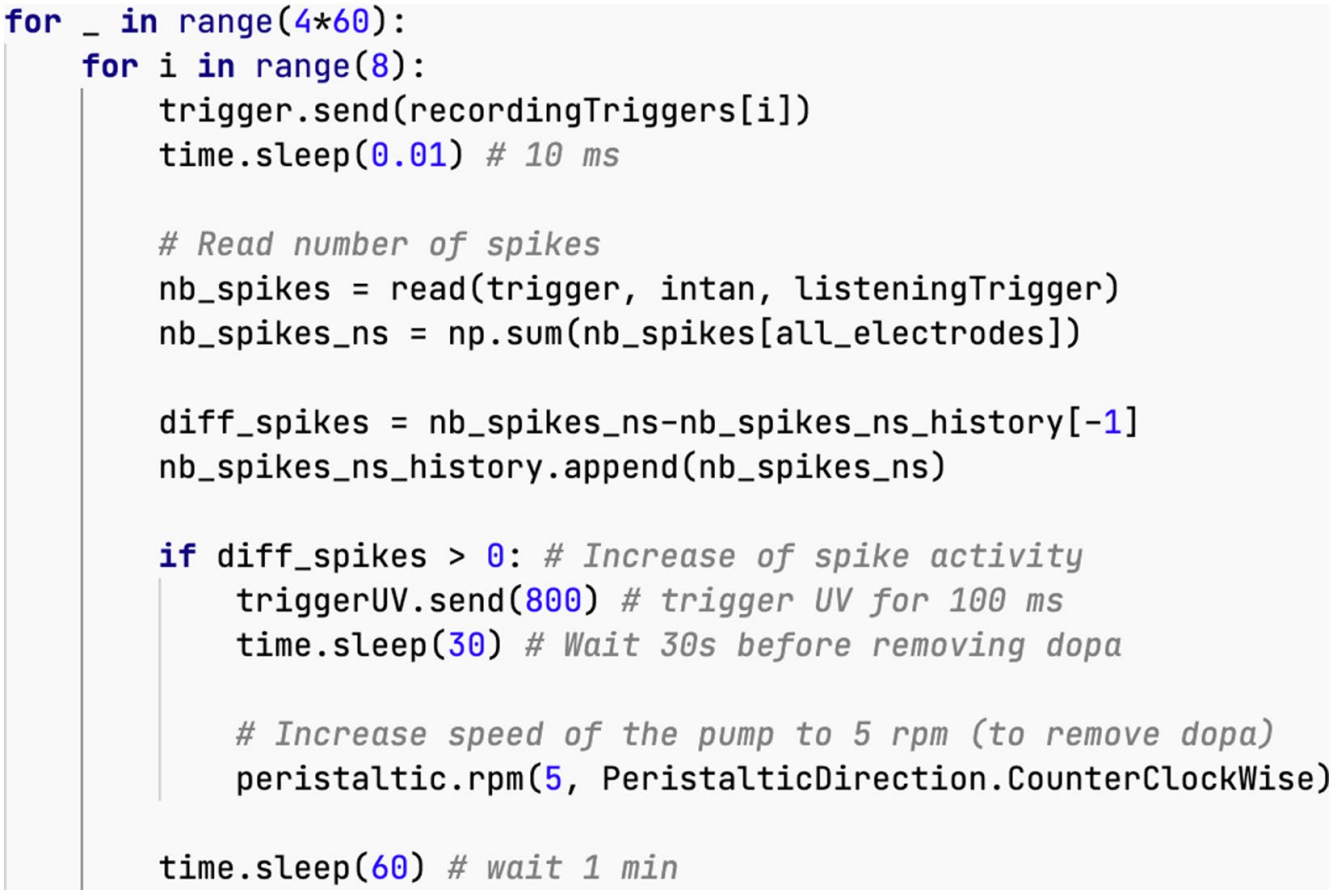


The graph in Figure 7D shows the variation in the number of spikes elicited during the execution of the script above across 5 h. A general increase in the number of elicited spikes can be observed. However, it is obviously not possible to establish causality between the closed-loop strategy and the observed increase with this single experiment alone. The primary purpose of this closed-loop experiment is to demonstrate the flexibility offered by the Neuroplatform.

## 6 External users of the Neuroplatform

Access to the Neuroplatform is freely available for research purposes. For researchers lacking lab infrastructure, the Neuroplatform provides the capability to conduct real-time experiments on biological networks. Additionally, it allows others to replicate results obtained in their own lab. The database is shared between all research groups, however the Python scripts and Jupyter Notebooks are in private sections.

In 2023, 36 academic groups proposed research projects, of which 8 were selected. At the time of writing, 4 of these have already yielded some results:

- University Côte d’Azure, CNRS, NeuroMod Institute and Laboratoire JA Dieudonné: investigates the functional connectivity of FO and how electrical stimulation can modify it.
- University of Michigan, investigates stimulation protocols that induce global changes in electrical activity of a FO.
- Free University of Berlin, investigates stimulation protocols that induce changes in the electrical activity of a FO. Additionally, this research employs machine learning tools to extract information from neural firing patterns and to develop well-conditioned responses. Moreover, it utilizes both shallow and deep reinforcement learning techniques to identify optimal training strategies, aiming to elicit reproducible behaviors in the FO.
- University of Exeter, Department of Mathematics and Statistics, Living Systems Institute, investigates storing and retrieving of spatiotemporal spiking patterns, using closed-loop experiments that combine mathematical models of synaptic communication with the Neuroplatform.
- Lancaster University Leipzig and University of York: characterizes computational properties of FOs under the reservoir computing model, with a view to building low-power environmental sensors.
- Oxford Brookes University, School of Engineering, Computing and Mathematics: investigating the properties of emerging dynamics and criticality within neural organizations using the FOs.
- University of Bath, ART-AI, IAH: using the free energy principle and active inference to study the learning capabilities of neurons, embodied in a virtual environment.
- University of Bristol: stimulating of FOs based on data gathered from an artificial tactile sensor. Use machine learning techniques to interpret the FO’s output, investigating their ability to process real-world data.

## 7 Discussion and conclusion

The Neuroplatform has now been operational 24/7 for the past 4 years. During this time, the organoids on the MEA have been replaced over 250 times. Considering that we place at least 4 organoids per MEA, and change all the organoids simultaneously, this amounts to testing over 1,000 organoids. Initially, their lifetime was only a few hours, but various improvements, especially related to the microfluidics setup, have extended this to up to 100 days in best cases. It is important to note that the spontaneous activity of the organoids can vary over their lifetime, a factor that must be taken into consideration when conducting experiments (Wagenaar et al., 2006). Additionally, we observed that the minimum current required to elicit spikes, computed using the method described in section 5.2, is increasing over the lifetime of the organoid. This phenomenon may be linked to an impedance increase caused by glial encapsulation (Salatino et al., 2017).

The 24/7 recording strategy as described in section 4.2, results in the constant growth of the database. As of this writing, its size has reached 18 terabytes. This volume encompasses the recording of over 20 billion individual action potentials, each sampled at a 30 kHz resolution for 3 ms. This extensive dataset is significant not only due to its size but also because it was all recorded in a similar *in-vitro* environment, as described in section 3.2. We are eager to share this data with any interested research group.

## 8 Future extensions

In the future, we plan to extend the capabilities of our platform to manage a broader range of experimental protocols relevant to wetware computing. For example, we aim to enable a remote control over the injection of specific molecules into the medium, facilitating remote experiments that involve pharmacological manipulation of neuronal activity. This expansion will provide additional degrees of freedom for the automatic optimization of parameters influencing neuroplasticity.

Currently, as detailed in Chapter 2, only one differentiation protocol is used for generating organoids. We plan to introduce additional types of organoid generation protocols soon, with the aim of exploring a broader range of possibilities.

Although 32 research groups requested to access to the Neuroplatform, our current infrastructure only allows us to accommodate 7 groups, considering our own research needs as well. We are in the process of scaling-up the AC/DC hardware system to support more users simultaneously. Additionally, we are currently limited to executing close-loop algorithms for neuroplasticity on one single FO, as these algorithms require sending in real-time adapted simulation signals to each FO. Our software is being updated to run closed-loops in parallel on up to 32 FO.

## 9 Methods

### 9.1 Brain organoid generation

Human forebrain organoids were originated as described in Govindan et al. (2021). Briefly, Human Neural Stem Cells derived from the human induced pluripotent stem (hiPS) cell line (ThermoFisher), were plated in flasks coated with CellStart (Fisher Scientific) and amplified in Stempro NSC SFM kit (ThermoFischer) complete medium: KnockOut D-MEM/F12, 2 mM of GlutaMAX, 2% of StemPro Neural supplement, 20 ng/mL of Human FGF-basic (FGF-2/bFGF) Recombinant Protein, and 20 ng/mL of EGF Recombinant Human Protein (Fisher Scientific). Cells were then detached with StemPro^™^ Accutase (Gibco) and plated in p6 at the concentration of 250,000 cells/well. The plates were sealed with breathable adhesive paper and leads, placed on an orbital shaker at 80 rpm, and culture for 7 days at 37°C 5% CO2. After one week the newly formed spheroids were put in differentiation medium I (Diff I), containing DMEM/F-12, GlutaMAX^™^ supplement (Gibco), 2% BSA, 1X of Stempro® hESC Supplement, 20 ng/mL of BDNF Recombinant Human Protein (Invitrogen), 20 ng/mL of GDNF Recombinant Human Protein (Gibco), 100 mM of N6,2′-O-Dibutyryladenosine 3′,5′-cyclic monophosphate sodium salt, and 20 mM of 2-Phospho-L-ascorbic acid trisodium salt. After one week, brain spheroids were put in differentiation medium II (Diff II) made of 50% of Diff I and 50% of Neurobasal Plus (Invitrogen). After 3 weeks of culture in Diff II, brain organoids were plated in Neurobasal Plus and kept in the orbital shaker until the transfer on the MEA. Medium was change once per week.

### 9.2 Electron microscopy analysis of FOs

Mature FOs were fixed in 2.5% Glutaraldehyde in 0.1 M phosphate buffer pH 7.4, at RT. After 24 h the samples were processed as described in Cakir et al. (2019) at the Electron Microscopy Facility of University of Lausanne. The whole FO images were acquired with Quanta FEG 250 Scanning Electron Microscope.

### 9.3 Transfer of FOs on MEA

MEA connected with the microfluid system was moved from the incubator and placed on a 12.3-megapixel camera system (with an optical lens of 16 mm of focal, giving a magnification power of 21x) inside the cell culture hood. The lid was removed to access the top of the liquid/air interface. Sterile Hydrophilic PTFE MEMBRANE Hole ‘confetti’ (diameter 2.5 mm, diameter of the hole 0.7 mm) (HEPIA) were positioned on top of each electrode and left there 2 min to absorb the medium. FOs were collected from the plate using wide bore pipette tips (Axygen) and placed in the middle of confetti, in a 10 μL drop of medium. The position of the organoids was adjusted with the help of sterile forceps. After all the organoids were put on place, the chamber was covered with the plate sealer Greiner Bio-One^™^ BREATHseal^™^ Sealer (Fisher Scientific), and with the MEA lid. MEA containing the organoids were placed immediately back in the cell incubator and were ready to be used for recording and stimulation. A similar procedure was used for the positioning of organoids on MCS MEA (60MEA200/30iR-Ti). In this case the Hydrophilic PTFE MEMBRANE was not used and organoids were directly laid on the electrodes in a 30 μL drop of medium. Recording of organoid activity was performed immediately afterwards.

### 9.4 System design and assembly

Cell culture media was stored in a 50 mL Falcon tube with a multi-port delivery cap (ElveFlow) and stored at 4°C. Each reservoir delivery cap contained a single 0.8 mm ID × 1.6 mm OD PTFE tubing (Darwin Microfluidics), sealed by a two-piece PFA Fittings and ferrule threaded adapter (IDEX), extending from the bottom of the reservoir to an inlet port on the 4-port valve head of the RVM Rotary Valve (Advance Microfluidics SA). Sterile air is permitted to refill the reservoir through a 0.22-μm filter (Milian) fixed to the cap to compensate for syringe pump medium withdrawal. A similar PTFE tubing and PFA Fittings and adapters were used to connect the syringe pump to the 4-port valve head of the RVM Rotary Valve (Advance Microfluidics SA). Each PTFE tubing coming from the distribution valve connects with a 50 mL falcon tube inside the cell culture incubator (Binder) and to a borosilicate glass bottle (Milian) to collect discarded cell culture medium.

A secondary microfluid system made of 0.8 mm ID × 1.6 mm OD PTFE tubing, were used to connect each 50 mL falcon tube inside the cell culture incubator with its own MEA (HEPIA). The connection was through a precise peristaltic pump BT100-2 J (Darwin Microfluidics) containing 10 rollers. A compute module (Raspberry Pi 4) controlled the peristaltic pump and the Rotary Valve, through a custom application program interface (API), using RS485 interface and RS-232 interface, respectively. A Fluigent flow-rate sensor connected via USB to the Raspberry Pi 4 allowed the monitoring of the flow rate inside the microfluidic system between the peristaltic pump and the MEA. Python was used to develop the software required to carry out automation protocols.

### 9.5 Uncaging of dopamine

Carboxynitroveratryl (CNV)-caged dopamine (Tocris Bioscience) was dissolved in Neurobasal Plus at the concentration of 1 mM, and injected in the fluidic system. After 3 h from the injection, the uncaging experiment started as described in paragraph 5.3. UV Silver-LED fiber-coupled LED (Prizmatix) was used to uncage the dopamine at the wavelength of 365 nm for 800 ms each time.

## Data availability statement

The raw data supporting the conclusions of this article will be made available by the authors, without undue reservation.

## Ethics statement

Ethical approval was not required for the studies on humans in accordance with the local legislation and institutional requirements because only commercially available established cell lines were used.

## Author contributions

FJ: Writing – original draft, Writing – review & editing. MK: Writing – original draft, Writing – review & editing. J-MC: Writing – original draft, Writing – review & editing. FB: Writing – original draft, Writing – review & editing. EK: Writing – original draft, Writing – review & editing.
